# Supplementary material for: Pitfalls in methods to study colocalization of nanoparticles in mouse macrophage lysosomes
Source: J Nanobiotechnology. 2022 Oct 29;20:464. doi: 10.1186/s12951-022-01670-9 (PMC9618187; doi:10.1186/s12951-022-01670-9)
Supplement: Supplementary file 1 — Additional file 1: Figure S1A. Comparison of the absorbance and emission spectra of the different LysoTracker probes. Figure S1B. Comparison of the emission spectra of the different (nano)particles. Figure S1C. Comparison of the absorbance spectra of the different (nano)particles. Figure S2. Confocal microscopy images of different LysoTracker probes. Figure S3. Transmission electron micrographs (TEM) of the different silica (nano)particles. Figure S4A. Size distributions of 59 nm SiO2-BDP FL NP and 59 nm SiO2-RhoB NP measured by dynamic light scattering. Figure S4B. Size distributions of 119 nm SiO2-Cy5 particles and 920 nm SiO2-Cy5 particles measured by dynamic light scattering. Figure S5A and S5B. Representative images of individual cells from different experiments for identification and contour with the corresponding histogram of the complete image acquired for each individual channel (nanoparticles and lysosomes). Figure S6. Representative bright field image of live cells (A) and fluorescent microscopy image of fixed cells (B) showing a population of individual cells used for colocalization analysis. Figure S7. Representative images of LysoTracker Red probe and Lamp-2 stainings in a single cell. Figure S8. Comparison of Pearson’s and Manders’ coefficients between live and fixed cells. Figure S9. Comparison of raw integrated intensity between live and fixed cells. Video S1. Continuous live cell imaging of J774A.1 cells. Script S1. Raw integrated densities. Script S2. Colocalization analysis. [file 12951_2022_1670_MOESM1_ESM.docx]

**Supplementary information**

**
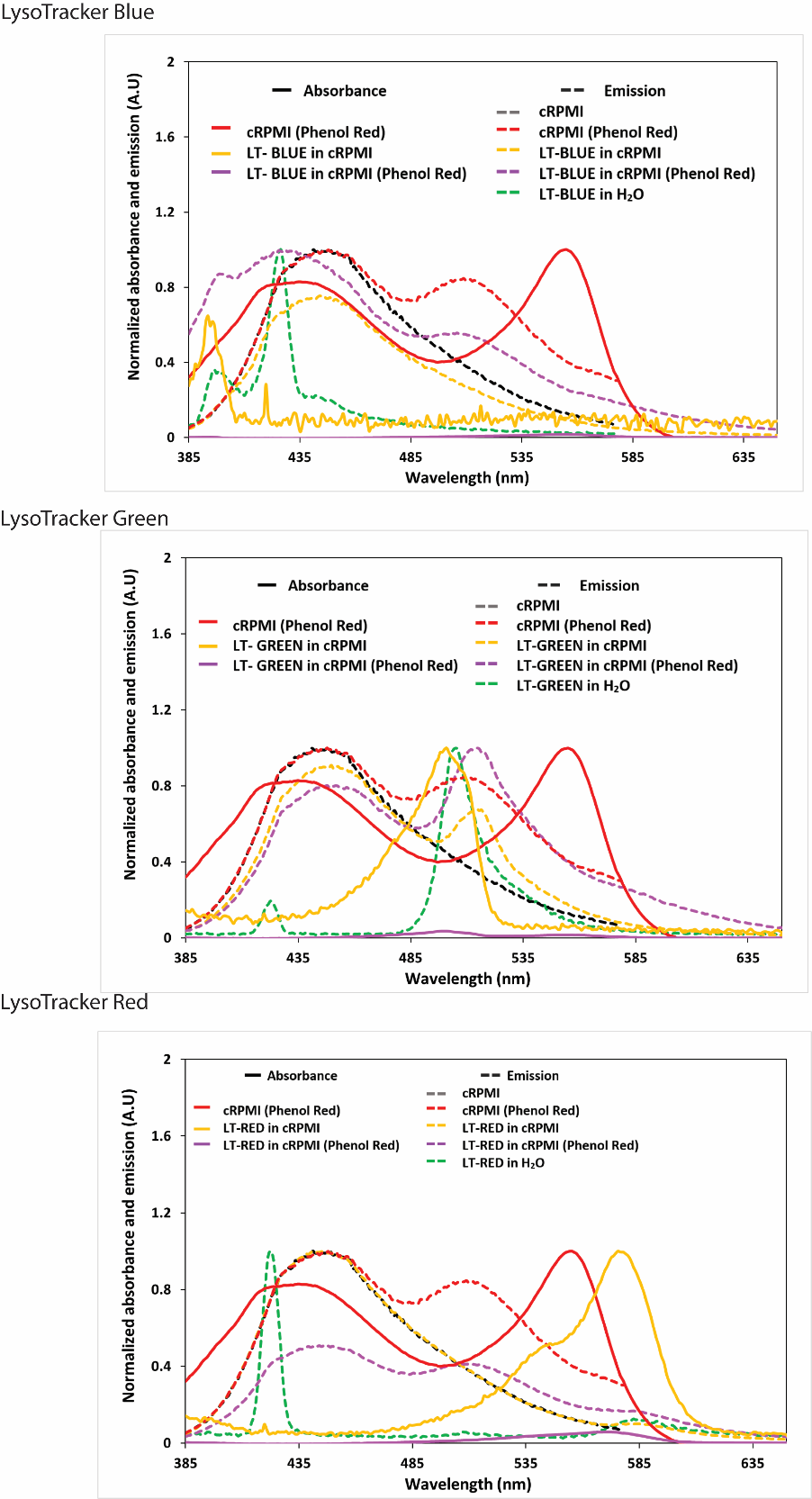
**

**Figure S1A.** Comparison of the absorbance and emission spectra of the different LysoTracker (LT) probes, in cRPMI with or without phenol red and the control emission spectra in Milli-Q water. Lines: absorbance, dash lines: emission.

Note: The UV-Vis Absorption Spectroscopy was carried out with a Jasco V-670 spectrophotometer. Quartz cuvettes (1 cm, science outlet) were used. The fluorescence Spectroscopy measurements were recorded with a Horiba Fluorolog 3 spectrometer equipped with a 450 W Xenon light source for excitation.


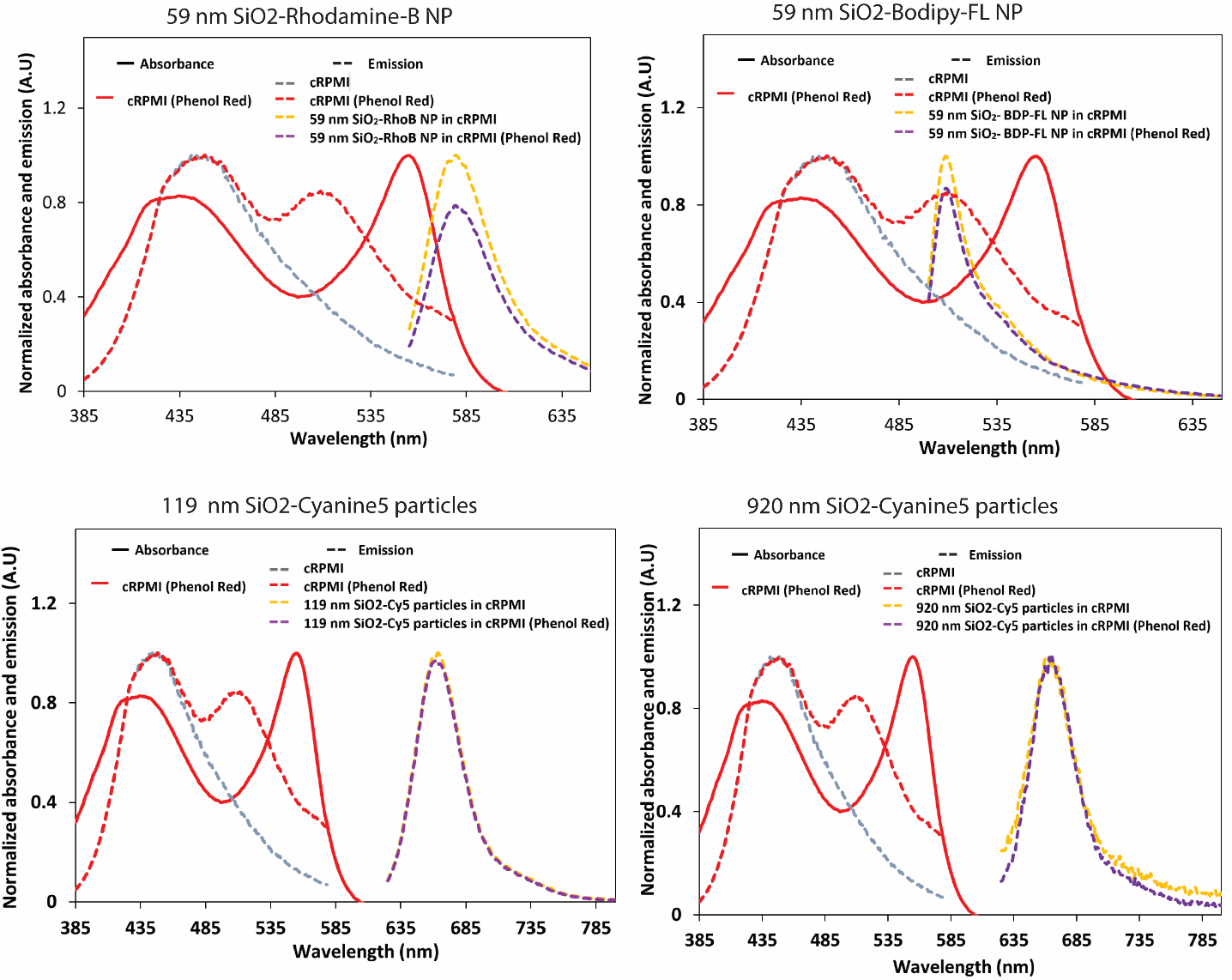


**Figure S1B.** Comparison of the emission spectra of the different (nano)particles in cRPMI with and without phenol red and the absorbance of cRPMI with phenol red. Lines: absorbance, dash lines: emission.

For the corresponding data to (nano)particles we measured the emission of (nano)particles instead of the pure dye. Since all the experiments were performed with the (nano)particles labeled with a specific dye, the microscopy fluorescent signals will correspond to the fluorophores that are incorporated into the (nano)particles. This makes the measurement closer to the real experimental conditions.


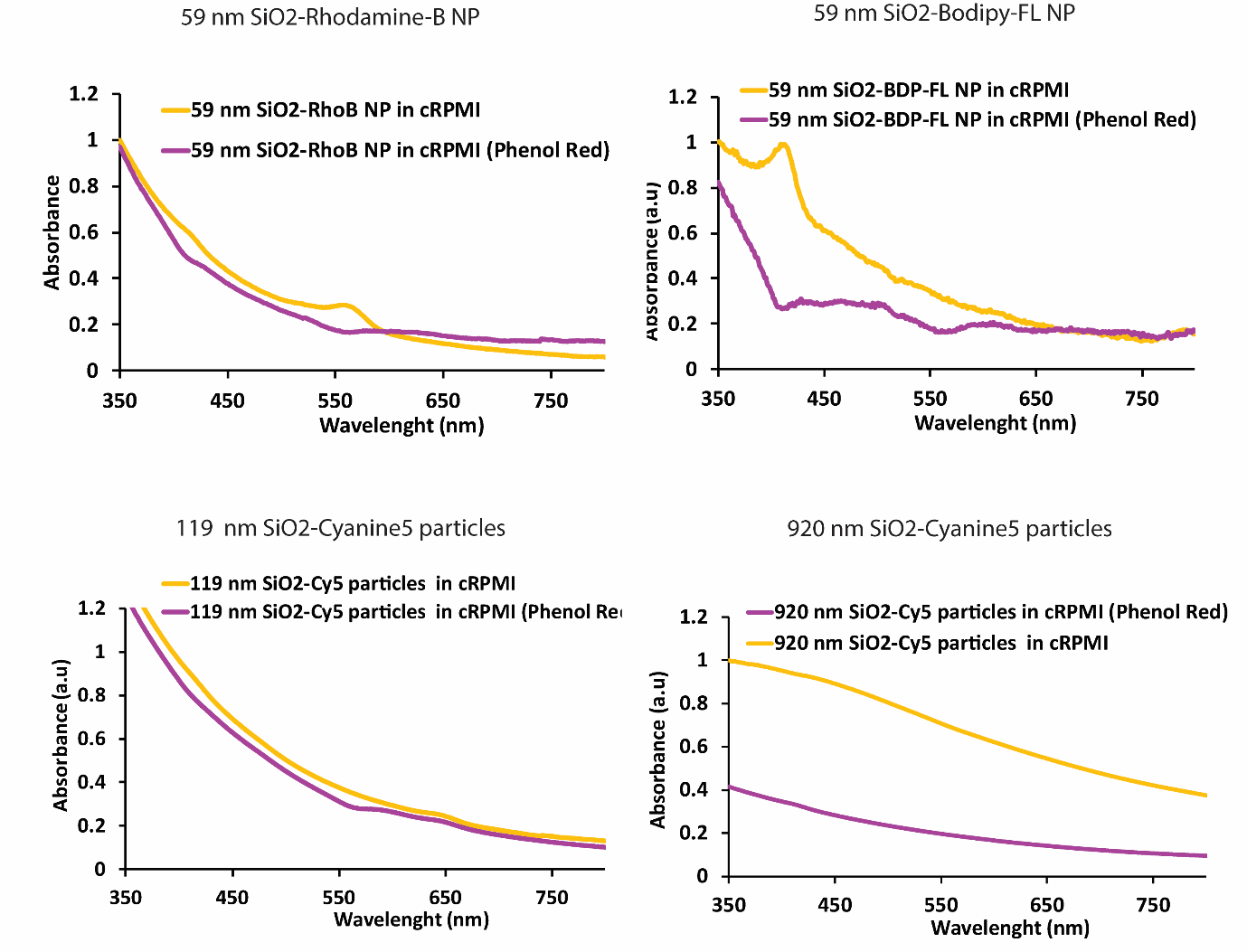


**Figure S1C.** Comparison of the absorbance spectra of the different (nano)particles in cRPMI with and without phenol red.

The absorbance spectra of the fluorophores in (nano)particles is dominated by the scattering of (nano)particles. This is the reason why the characteristic peak of the fluorophores is not strongly visualized. Nevertheless, the characteristic peak of the fluorophore is clearly visualized in the emission spectra in **Figure S1B**.


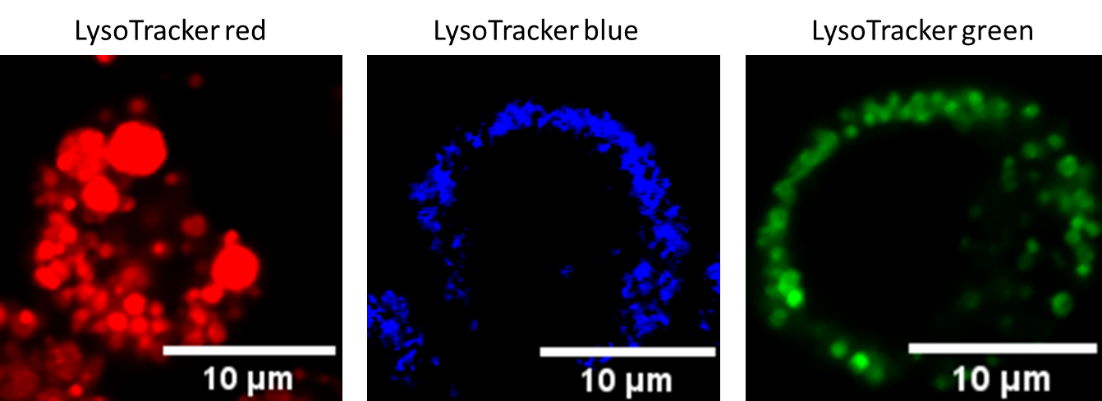


**Figure S2.** Confocal microscopy images representing different LysoTracker probes.


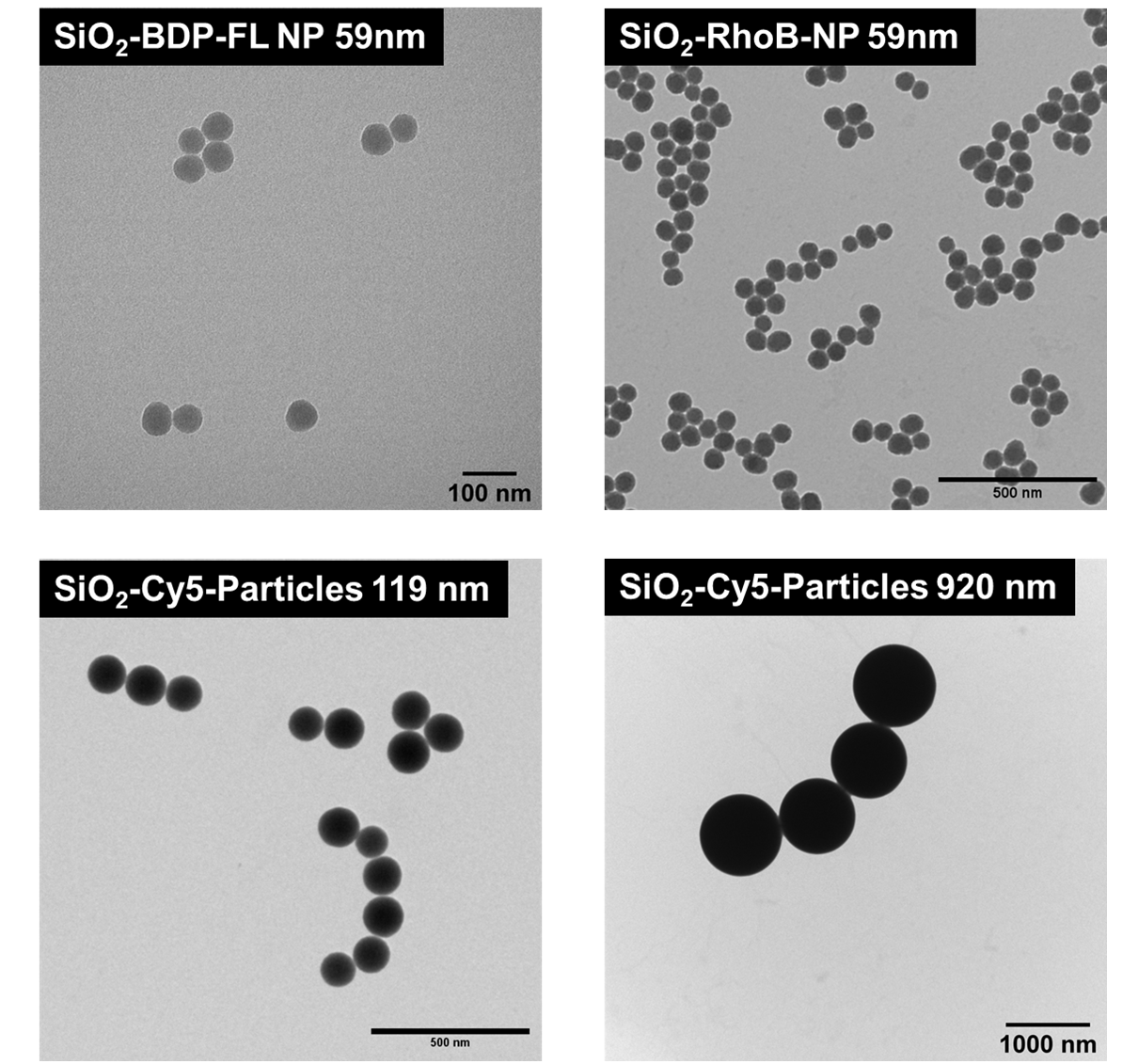


**Figure S3.** Transmission electron micrographs (TEM) of the different silica (nano)particles: 59 nm SiO_2_-BDP FL NP, 59 nm SiO_2_-RhoB NP, 119 nm SiO_2_-Cy5 particles and 920 nm SiO_2_-Cy5 particles.

*
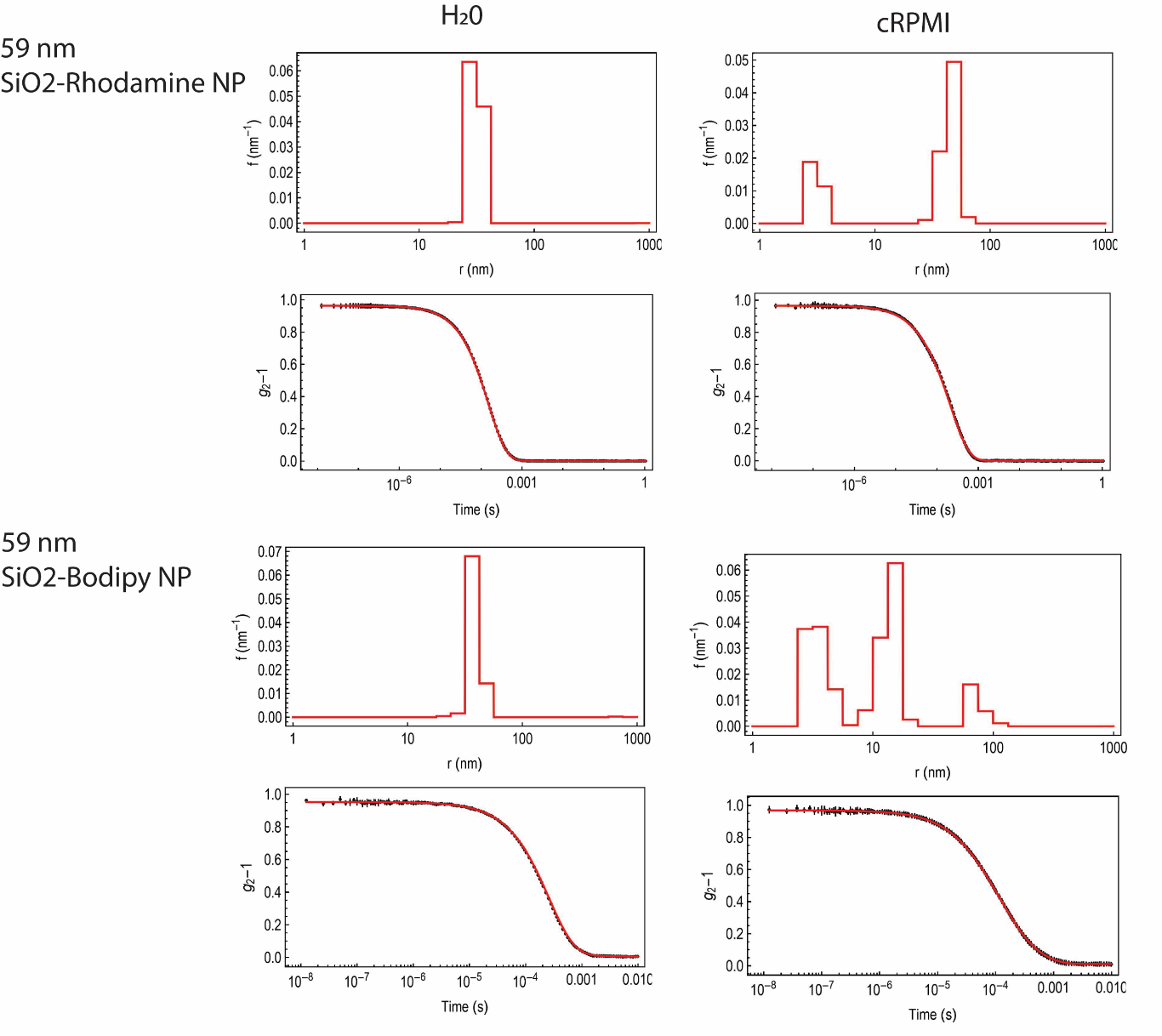
*

**Figure S4A.** Size distributions of 59 nm SiO_2_-BDP FL NP and 59 nm SiO_2_-RhoB NP measured by dynamic light scattering in Milli-Q water and cRPMI. Representative autocorrelation functions for the DLS results are included below each histogram.

**
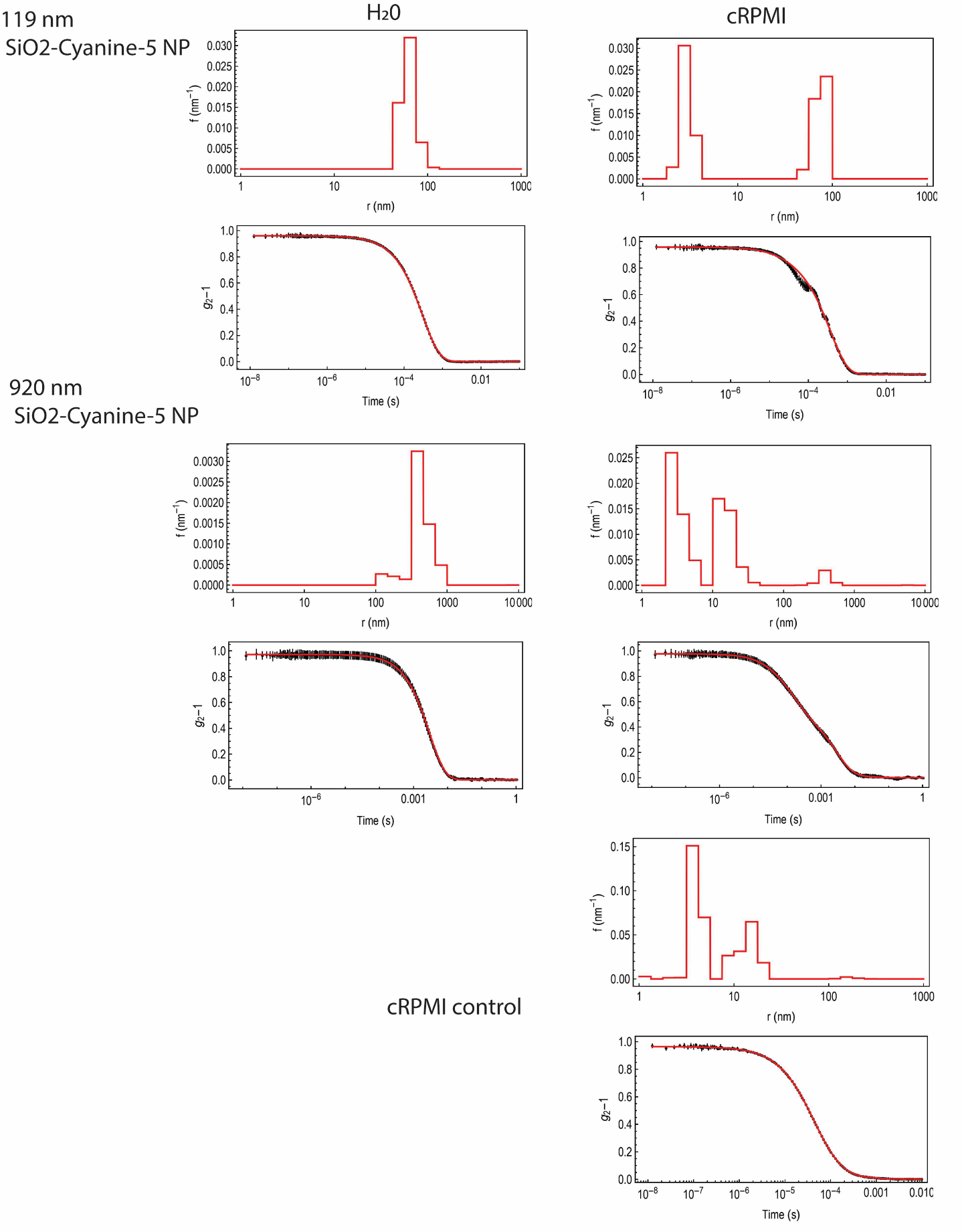
**

**Figure S4B.** Size distributions of 119 nm SiO_2_-Cy5 particles and 920 nm SiO_2_-Cy5 particles measured by dynamic light scattering in Milli-Q water and cRPMI. Representative autocorrelation functions for the DLS results are included below each histogram.


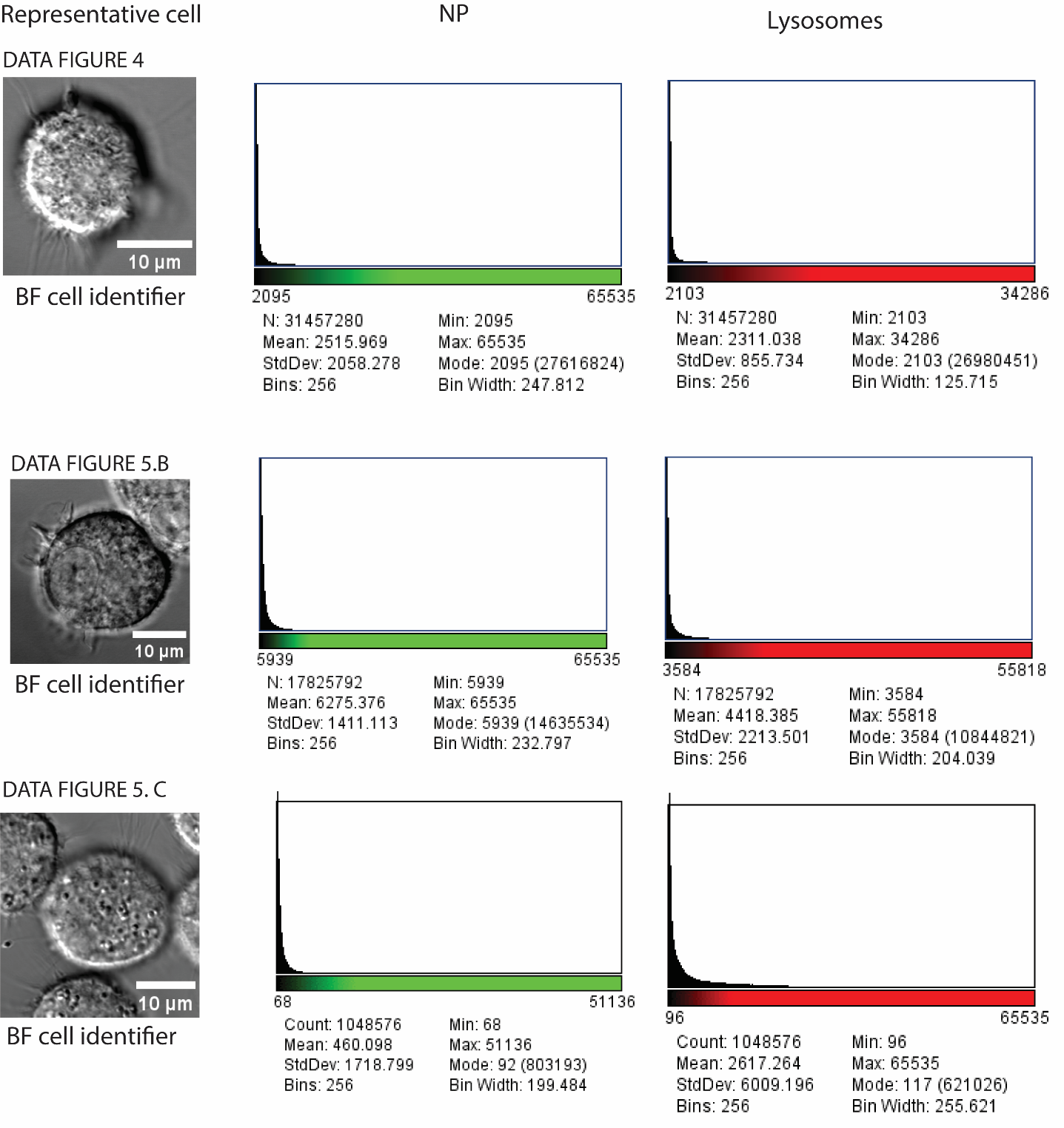
**Figure S5A.** Representative images of individual cells from different experiments for identification and contour. The corresponding histograms of the complete image acquired for each individual channel (nanoparticles and lysosomes) are shown to confirm that there was no pixel overexposure. BF: Bright field image.

*
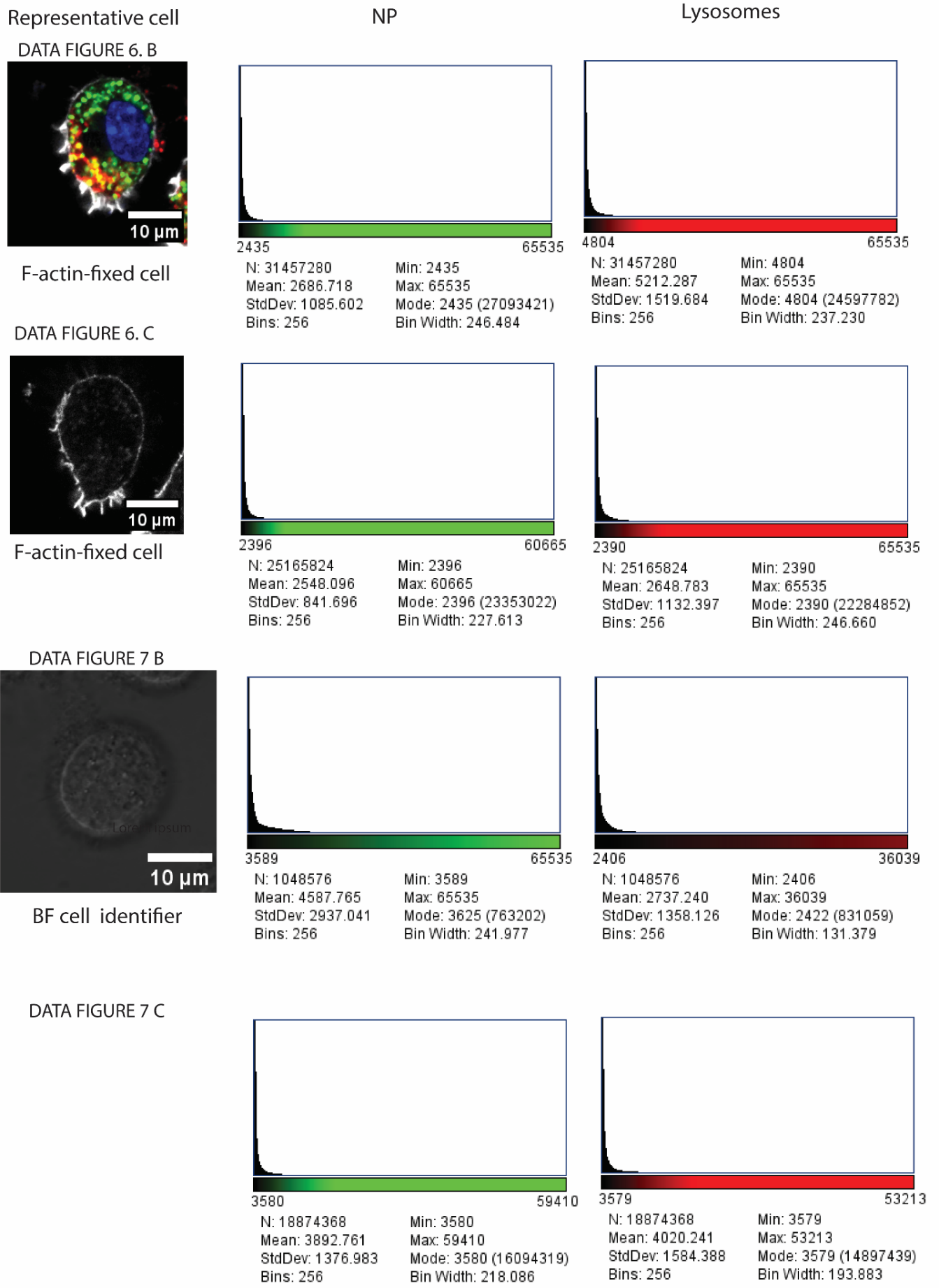
*

**Figure S5B.** Representative images of individual cells for identification and contour with the corresponding histogram of the complete image acquired for each individual channel (nanoparticles and lysosomes). BF: Bright field image, F-actin (shown in white).


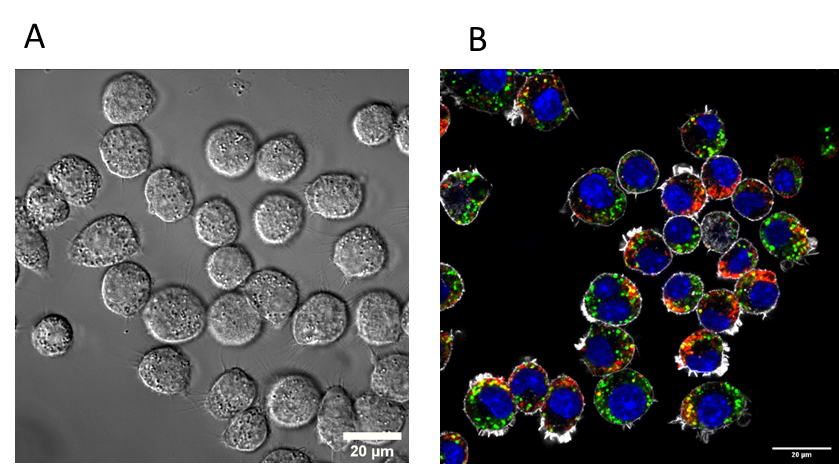


**Figure S6.** Representative bright field image of live cells **(A)** and fluorescent microscopy image of fixed cells **(B)** showing a population of individual cells used for colocalization analysis. Fixed cells were stained to identify cytoskeleton (F-actin; white), nuclei (DAPI; blue), NP (green) and lysosomes (LAMP-2, red).


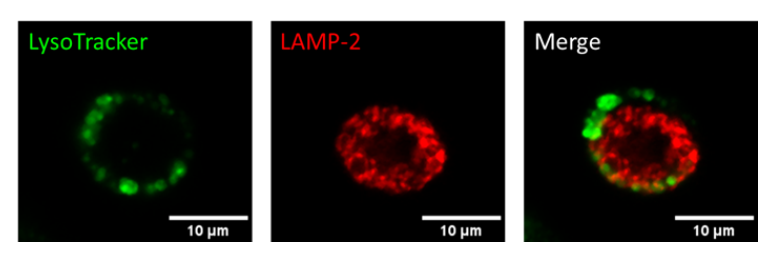


**Figure S7.** Representative images of LysoTracker Red probe and LAMP-2 staining in a single cell. Left: Lysosomes stained with LysoTracker Red probe (green), middle: Lysosomes stained with LAMP-2 antibody (red) and right: merged channels of LysoTracker Red probe and LAMP-2 of the same cell.

**
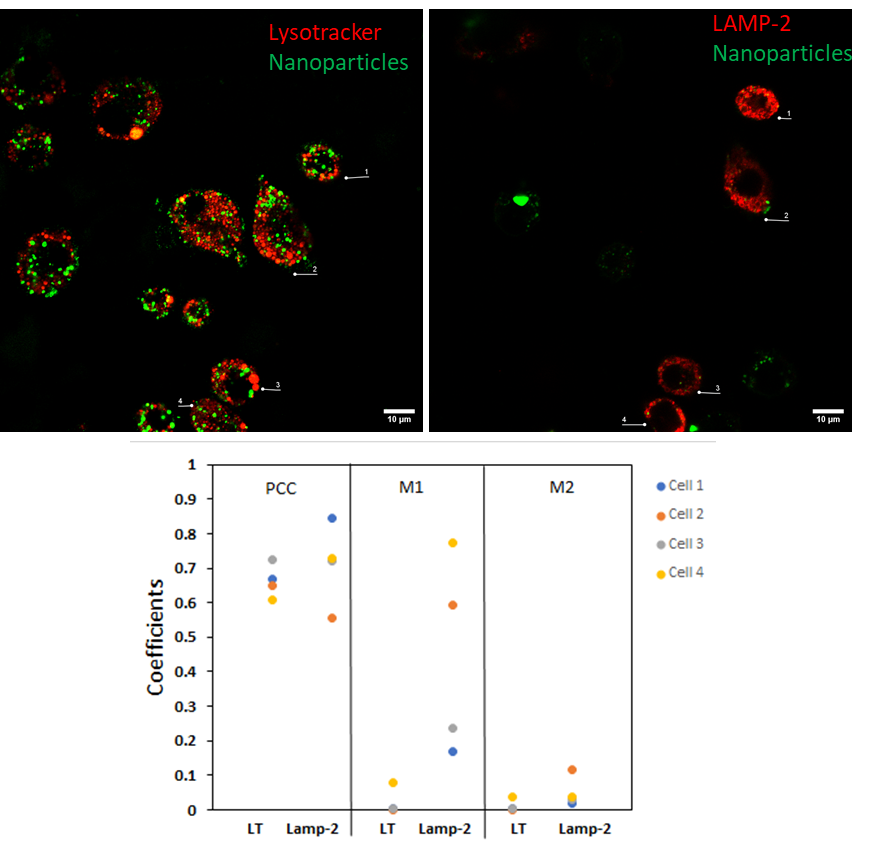
**

**Figure S8.** Comparison of Pearson’s and Manders’ coefficients between live and fixed cells. The cells were exposed to 59 nm SiO_2_ –BDP FL NP for 1h, stained with LysoTracker Red probe and imaged by CLSM. Then, the same sample was fixed, stained for LAMP-2 and imaged. A total of 4 cells were analyzed for comparision, each cell has a representative color as shown in the coefficients plot. There is a variability in colocalization coefficients between NP and lysosomes in individual cells. This data demonstrates that the coefficients from live and fixed cells cannot be directly compared. Additionally, comparing only Pearson’s correlation coefficients is not sufficient to confirm the distribution of the NP fraction in the lysosomes.

**
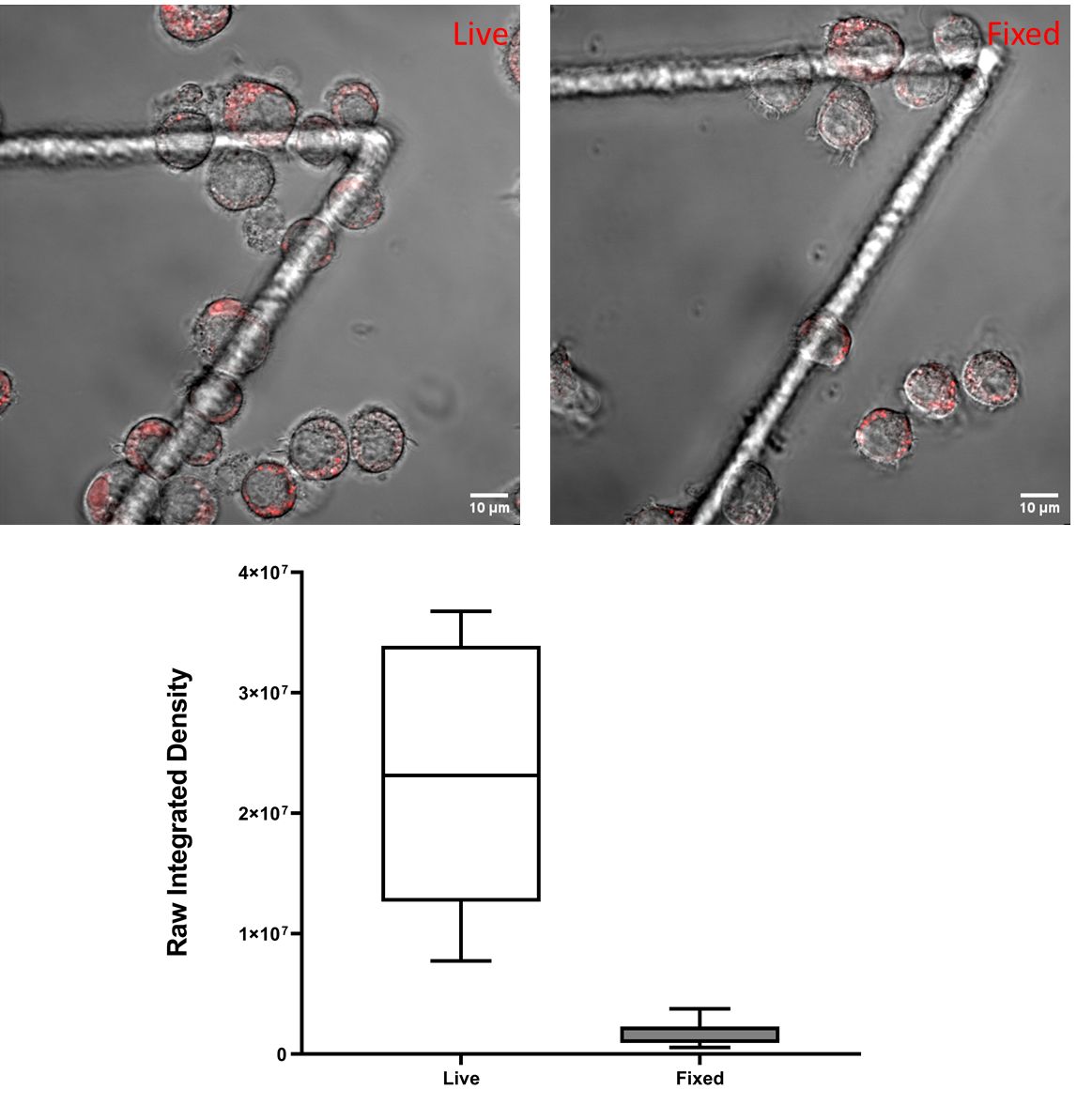
**

**Figure S9.** Comparison of raw integrated intensity between live and fixed cells. Top two images represent CLSM data from live cells (left) and fixed cells (right). The cells were stained with LysoTracker Red probe for 1h and imaged. The same cells were fixed and washed following a fixation protocol and imaged using the same settings. At the bottom, the boxplots show the raw integrated densities of live and fixed cells. The whiskers represent the standard deviations. Analysis was performed in 8 individual cells for each experiment. All the images were analyzed using ImageJ. There is a significant decrease in LysoTracker Red intensity in fixed cells, compared to live cells.

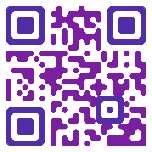


**Video S1**. Continuous live cell imaging of J774A.1 cells, stained with LysoTracker Red for 24 h, showing increased cell death over time.

**Script S1.** Raw integrated densities

It was done comparing 10 cells for each lysotracker using image J comparing the raw intensity densities and the mean gray values, selecting each cell with a mask using the next script and manual threshold:

//setTool("rectangle");

rename("source");

run("In [+]");

run("In [+]");

run("Duplicate...", "title=mask");

run("In [+]");

run("In [+]");

run("Median...", "radius=5");

setAutoThreshold("Default dark");

//run("Threshold...");

setOption("BlackBackground", true);

run("Convert to Mask");

run("Dilate");

run("Create Selection");

run("Make Inverse");

run("Make Inverse");

roiManager("Add");

roiManager("Select", 4);

roiManager("Select", 0);

roiManager("Select", 2);

selectWindow("source");

roiManager("Select", 0);

roiManager("Select", 2);

run("Measure");

**Script S2.** Colocalization analysis

**path1 = " ______________________ ";**

**timepoint= ______________________;**

**//channel 1 - NPS**

**ThresholdA = ______________________ ;**

**//channel 2 - LT**

**ThresholdB = ______________________ ;**

**Path2 = " ______________________ /JACOP/";**

**filename= " ______________________.tif";**

**// IMPORTANT: DO THE FOLLOWING BEFORE EACH MEASURMENT**

**//Check the channels order>**

**//channel 1 - NPS**

**//channel 2 - LT**

**// ADJUST THE ORDER IF IT IS NEEDED AND CHECK EACH CHANNEL THRESHOLD**

**// #########**

**// OPEN DATA**

**// #########**

**open(filename);**

**selectWindow(filename);**

**rename("raw");**

**run("Select None");**

**roiManager("reset")**

**roiManager("Open", path1 +"/Roi.zip");**

**run("Colors...", "foreground=black background=black selection=yellow");**

**setBatchMode(true);**

**print("\\Clear");**

**// #######################**

**// CYCLE THROUGH EACH CELL**

**// #######################**

**for (cell = 0; cell < roiManager("count"); cell++){**

**//-------------------------------------------**

**// Cut out the cell and delete the background**

**//-------------------------------------------**

**selectWindow("raw");**

**run("Enhance Contrast", "saturated=0.35");**

**run("Select None");**

**roiManager("Select", cell);**

**run("Duplicate...", "duplicate title=[cell 1] frames="+timepoint);**

**run("Make Inverse");**

**run("Fill", "stack");**

**run("Select None");**

**rename("data");**

**run("Split Channels");**

**//------------------------**

**// Run the colocalization**

**//------------------------**

**//without Costes randomizations**

**run("JACoP ", "imga=C1-data imgb=C2-data thra="+ThresholdA+" thrb="+ThresholdB+" pearson overlap mm ccf=20 cytofluo ica costesrand=2-1-1000-0.001-0-false-true-true");**

**// with Costes randomizations (takes long)**

**//run("JACoP ", "imga=C1-data imgb=C2-data thra="+ThresholdA+" thrb="+ThresholdB+" pearson overlap mm costesthr ccf=20 cytofluo ica costesrand=2-1-1000-0.001-0-false-true-true");**

**//-----------------------------------------------------------**

**// Check the resulting windows, save them and then close them**

**//-----------------------------------------------------------**

**if (isOpen("Costes' threshold C1-data and C2-data")){**

**selectWindow("Costes' threshold C1-data and C2-data");**

**saveAs("Tiff", Path2 +cell+"_Costes' threshold C1-data and C2-data.tif");**

**close();**

**}**

**if (isOpen("Costes' mask")){**

**selectWindow("Costes' mask");**

**saveAs("Tiff", Path2 +cell+"_Costes' mask.tif");**

**close();**

**}**

**if (isOpen("Van Steensel's CCF between C1-data and C2-data")){**

**selectWindow("Van Steensel's CCF between C1-data and C2-data");**

**Plot.setStyle(3, "blue,none,2.0,Line");**

**saveAs("Tiff", Path2 +cell+"_Van Steensel's CCF between C1-data and C2-data.tif");**

**close();**

**}**

**if (isOpen("Cytofluorogram between C1-data and C2-data")){**

**selectWindow("Cytofluorogram between C1-data and C2-data");**

**Plot.setStyle(0, "black,none,2.0,Dot");**

**saveAs("Tiff", Path2 +cell+"_Cytofluorogram between C1-data and C2-data.tif");**

**close();**

**}**

**if (isOpen("ICA A (C1-data)")){**

**selectWindow("ICA A (C1-data)");**

**Plot.setStyle(1, "black,none,2.0,Dot");**

**saveAs("Tiff", Path2 +cell+"_ICA A (C1-data).tif");**

**close();**

**}**

**if (isOpen("ICA B (C2-data)")){**

**selectWindow("ICA B (C2-data)");**

**Plot.setStyle(1, "black,none,2.0,Dot");**

**saveAs("Tiff", Path2 +cell+"_ICA B (C2-data).tif");**

**close();**

**}**

**if (isOpen("Randomized images of C2-data")){**

**selectWindow("Randomized images of C2-data");**

**saveAs("Tiff", Path2 +cell+"_Randomized images of C2-data.tif");**

**close();**

**}**

**if (isOpen("Costes' method (C1-data & C2-data)")){**

**selectWindow("Costes' method (C1-data & C2-data)");**

**Plot.setStyle(3, "blue,none,3.0,Line");**

**Plot.setStyle(0, "white,none,1.0,Linehidden");**

**Plot.setStyle(1, "black,none,2.0,Circle");**

**saveAs("Tiff", Path2 +cell+"_Costes' method (C1-data & C2-data).tif");**

**close();**

**}**

**if (isOpen("C2-data")) {**

**selectWindow("C2-data");**

**saveAs("Tiff", Path2 +cell+"_C2-data.tif");**

**close();**

**}**

**if (isOpen("C1-data")) {**

**selectWindow("C1-data");**

**saveAs("Tiff", Path2 +cell+"_C1-data.tif");**

**close();**

**}**

**//------------------------------------------------------------------------**

**// Extract the coloc data from the Log window an post it in a result table**

**//------------------------------------------------------------------------**

**setResult("Cell", cell, "cell "+ cell);**

**logString = getInfo("Log");**

**selectWindow("Log");**

**saveAs("Text", Path2 +cell+"_Log.txt");**

**logString = getInfo("Log");**

**selectWindow("Log");**

**setResult("Pearsons correlation coefficient", cell, substring(logString, indexOf(logString, "Pearson's Coefficient:")+25, indexOf(logString, "Overlap Coefficient:")-2));**

**setResult("Overlap coefficient (no threshold)", cell, substring(logString, indexOf(logString, "Overlap Coefficient:")+23, indexOf(logString, "r^2=k1xk2:")-2));**

**setResult("k1 (no threshold)", cell, substring(logString, indexOf(logString, "r^2=k1xk2:")+14, indexOf(logString, "r^2=k1xk2:")+19));**

**setResult("k2 (no threshold)", cell, substring(logString, indexOf(logString, "r^2=k1xk2:")+23, indexOf(logString, "r^2=k1xk2:")+28));**

**setResult("Threshold A", cell, substring(logString, indexOf(logString, "Using thresholds (thrA=")+23, indexOf(logString, " and thrB=")));**

**logString = substring(logString, indexOf(logString, "r^2=k1xk2:")+28, lengthOf(logString));**

**setResult("Threshold B", cell, substring(logString, indexOf(logString, "thrB=")+5,indexOf(logString, "Overlap Coefficient:")-3));**

**logString = substring(logString, indexOf(logString, "Using thresholds ")+28, lengthOf(logString));**

**setResult("Overlap coefficient (with threshold)", cell, substring(logString, indexOf(logString, "Overlap Coefficient:")+23, indexOf(logString, "r^2=k1xk2:")-2));**

**setResult("k1 (with threshold)", cell, parseFloat(substring(logString, indexOf(logString, "r^2=k1xk2:")+14, indexOf(logString, "r^2=k1xk2:")+19)));**

**setResult("k2 (with threshold)", cell, parseFloat(substring(logString, indexOf(logString, "r^2=k1xk2:")+23, indexOf(logString, "r^2=k1xk2:")+28)));**

**setResult("Manders coefficient (without threshold) M1", cell, substring(logString, indexOf(logString, "Manders' Coefficients (original")+37, indexOf(logString, " (fraction of A overlapping B)")));**

**setResult("Manders coefficient (without threshold) M2", cell, substring(logString, indexOf(logString, " (fraction of A overlapping B)")+34, indexOf(logString, " (fraction of B overlapping A)")));**

**logString = substring(logString, indexOf(logString, "Manders' Coefficients (using threshold value of"), lengthOf(logString));**

**setResult("Manders coefficient (with threshold) M1", cell, substring(logString, indexOf(logString, "M1=")+3, indexOf(logString, " (fraction of A overlapping B)")));**

**setResult("Manders coefficient (without threshold) M2", cell, substring(logString, indexOf(logString, "M2=")+3, indexOf(logString, " (fraction of B overlapping A)")));**

**setResult("Van Steensel CCFmin", cell, substring(logString, indexOf(logString, "CCF min.:")+10, indexOf(logString, "(obtained")-1));**

**setResult("Van Steensel CCFmin peak value", cell, substring(logString, indexOf(logString, "dx=")+3, indexOf(logString, "CCF max.")-2));**

**logString = substring(logString, indexOf(logString, "CCF max.")-2, lengthOf(logString));**

**setResult("Van Steensel CCFmax", cell, substring(logString, indexOf(logString, "CCF max.:")+10, indexOf(logString, "(obtained")-1));**

**setResult("Van Steensel CCFmax peak value", cell, substring(logString, indexOf(logString, "dx=")+3, indexOf(logString, "Results for fitting")-3));**

**setResult("Cytofluorogram a", cell, substring(logString, indexOf(logString, "Cytofluorogram's parameters:")+32, indexOf(logString, "b: ")-3));**

**setResult("Cytofluorogram a", cell, substring(logString, indexOf(logString, "b: ")+3, indexOf(logString, "Correlation coefficient:")-3));**

**setResult("Cytofluorogram Correlation Coeffcient", cell, substring(logString, indexOf(logString, "Correlation coefficient: ")+25, indexOf(logString, "Li's")-2));**

**setResult("Li's ICQ", cell, substring(logString, indexOf(logString, "ICQ")+4, indexOf(logString, "Costes' randomization based colocalization:")-2));**

**setResult("Costes's R original ", cell, substring(logString, indexOf(logString, "r (original)")+13, indexOf(logString, "r (randomized)")-1));**

**setResult("Costes's R randomized ", cell, substring(logString, indexOf(logString, "r (randomized)")+15, indexOf(logString, "±")));**

**setResult("Costes's R original SD", cell, substring(logString, indexOf(logString, "±")+1, indexOf(logString, "(calculated from the fitted data")));**

**setResult("Costes's R P-value in %", cell,substring(logString, indexOf(logString, "(calculated from the fitted data")+42, indexOf(logString, "%")));**

**//------------------------**

**// Reset the log window**

**//------------------------**

**print("\\Clear");**

**selectWindow("Log");**

**run("Close" );**

**//-------------------------------------------**

**// End of cycle, goto next cell, if available**

**//-------------------------------------------**

**}**

**// #########**

**// CLOSE DATA**

**// #########**

**selectWindow("raw");**

**close();**
